# Supplementary material for: Prosocial decisions in naturalistic helping scenarios are predicted by cost-benefit tradeoffs and individual disposition
Source: Commun Psychol. 2025 Dec 20;4:2. doi: 10.1038/s44271-025-00371-x (PMC12770495; doi:10.1038/s44271-025-00371-x)
Supplement: Supplementary file 2 — Supplementary Information [file 44271_2025_371_MOESM2_ESM.pdf]

Prosocial Decisions in Naturalistic Helping Scenarios Are Predicted by Cost-Benefit Tradeoffs and Individual Disposition

Qianying Wu[1,2], Miao Song[1], Jackie Ayoub[1], David Dunning[3], Danyang Tian[1], and Ehsan Moradi-Pari[1]

1. Honda Research Institute USA, San Jose, USA
2. California Institute of Technology, Pasadena, USA
3. University of Michigan, Ann Arbor, Ann Arbor, USA

Supplementary Information

Supplementary Figures

Supplementary Figure 1. Participant recruitment and exclusion procedure

Supplementary Figure 2. WTH variance explained by each motivational dimension

Supplementary Figure 3. Exploratory factor analysis on the original motivation dimensions

Supplementary Figure 4. Parameter recovery

Supplementary Figure 5. Comparison between the binary decision model and the continuous rating model.

Supplementary Figure 6. Associations between willingness to help and demographic variables

Supplementary Figure 7. Associations among three spaces

Supplementary Figure 8. Decomposition of the decision similarity space

Supplementary Tables

Supplementary Table 1. Rating task questions

Supplementary Table 2. Model comparisons

Supplementary Table 3. Post-hoc pairwise comparisons of WTH across four quadrants.

Supplementary Table 4. Summary of self-reported additional motivations

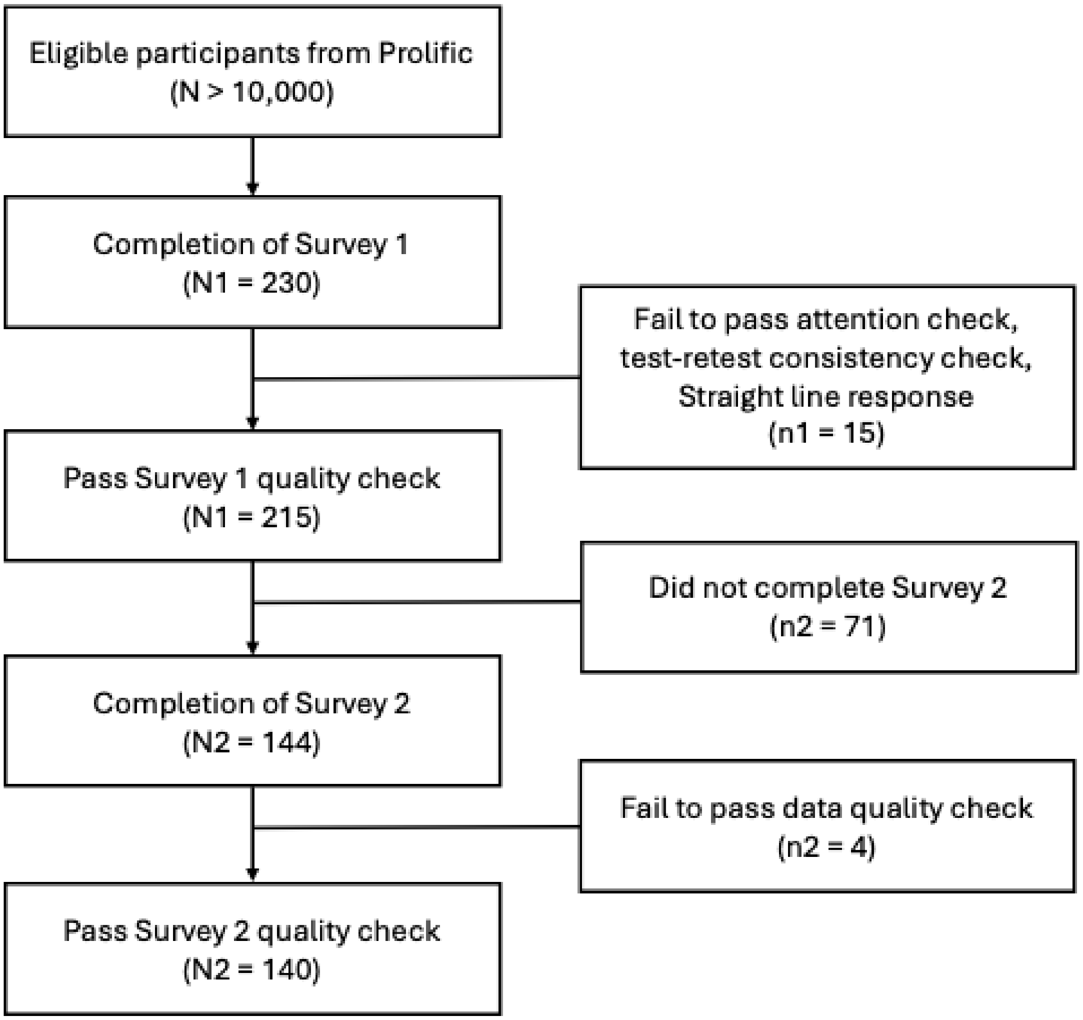

Supplementary Figure 1. Participant recruitment and exclusion procedure. The number of participants included and excluded at each recruitment and analysis step. N denotes the number of participant inclusion, and n denotes the number of participant exclusion.

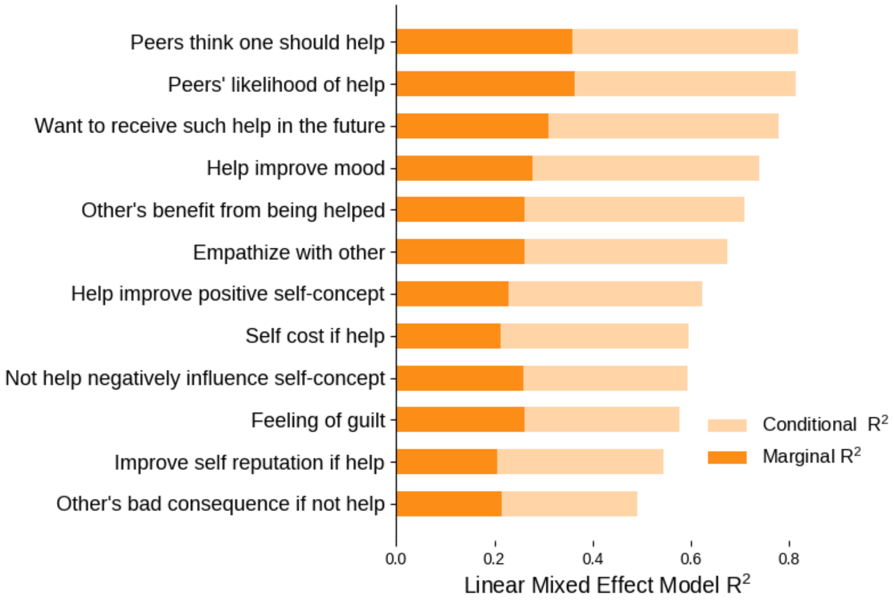

Supplementary Figure 2. WTH variance explained by each motivational dimension. Explained variance was assessed using marginal R^2 (for fixed effect of motivation, dark orange) and conditional R^2 (for both fixed effect and random effect, light orange). The bars are superimposed, thus the magnitude of each R^2 is shown in its corresponding x axis values.

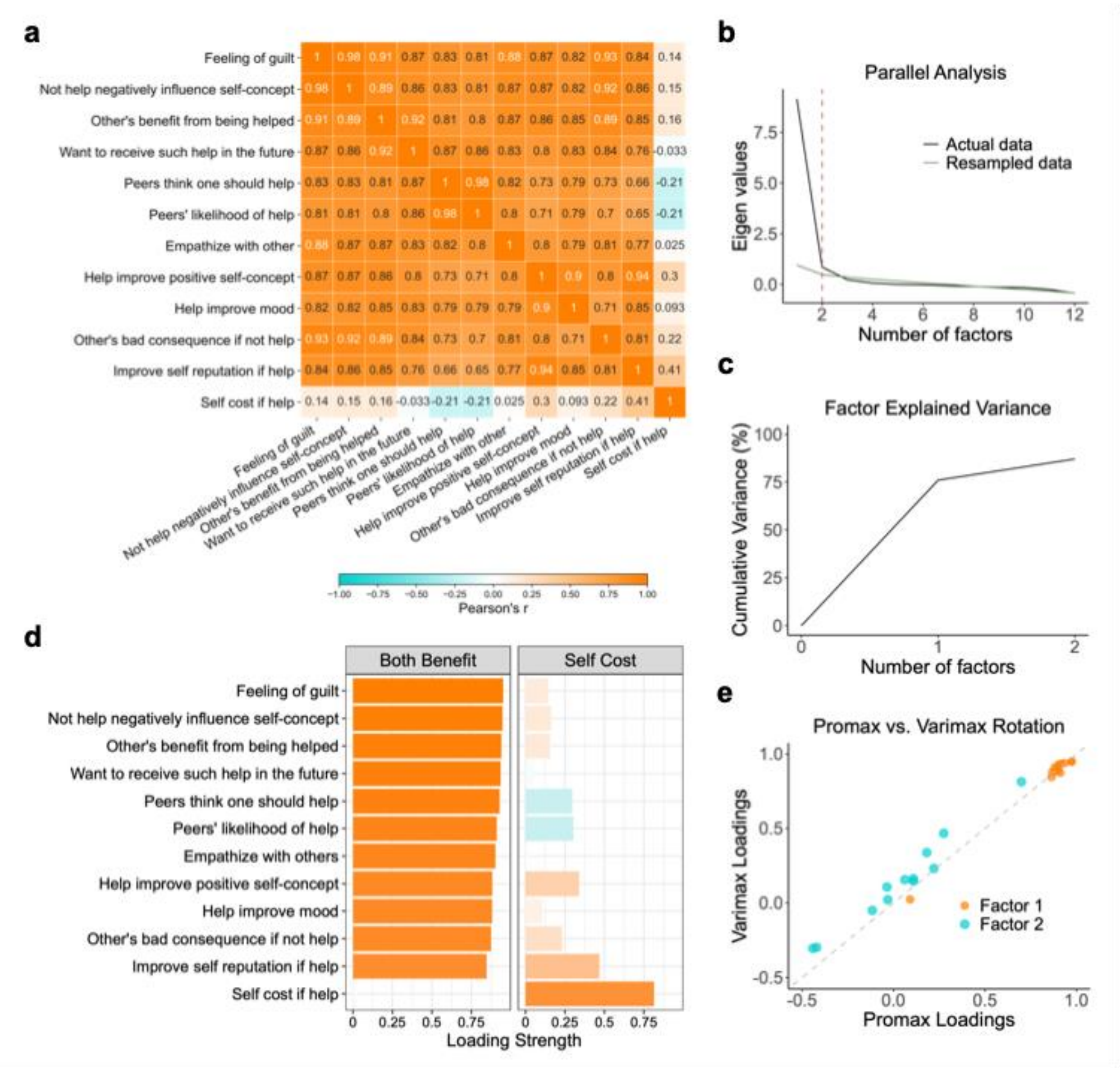

Supplementary Figure 3. Exploratory factor analysis on the original motivation dimensions. (a) Correlations among the ratings of 12 original motivation dimensions. Orange indicates positive correlations, and blue indicates negative correlations. The color intensity is proportional to Pearson's r (dof = 98). (b) Parallel analysis. The optimal number of factors is 2, determined by the maximum number before the scree plots from the actual data and resampled simulated data intersect. (c) Cumulative variance explained by each factor. Factors were sorted in the descending order of their explained variance. (d) Loadings of the motivation dimensions on identified factors. Orange indicates positive loadings, and blue indicates negative loadings. The color intensity is proportional to the loading strength. (e) Comparison of factor loadings between promax and varimax rotation results. Each dot represents a motivation dimension.

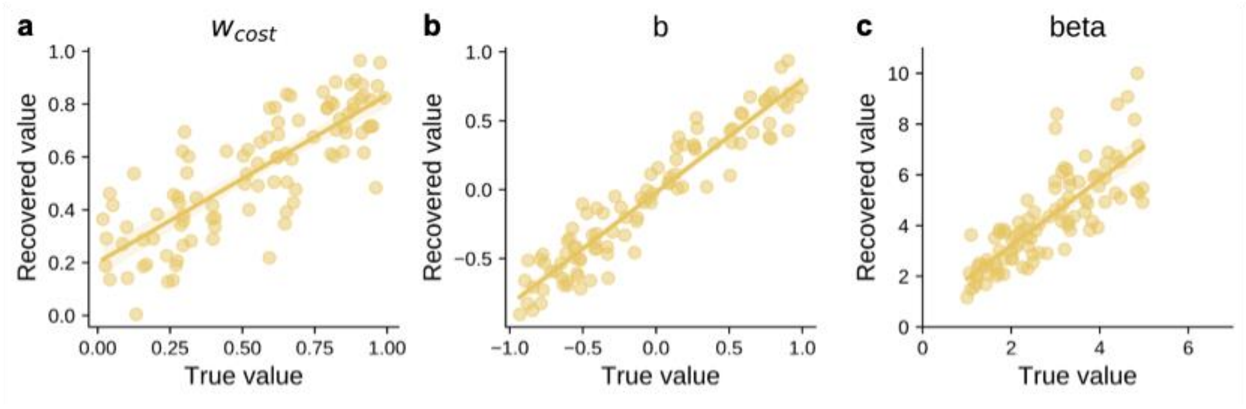

Supplementary Figure 4. Parameter recovery. Comparisons between 100 sets of true model parameter values that were used to generate simulated datasets and the parameter values fitted from the simulated datasets. (a) Parameter w_{cost}. (b) Parameter b. (c) Parameter β. Each dot represents one set of parameter simulation.

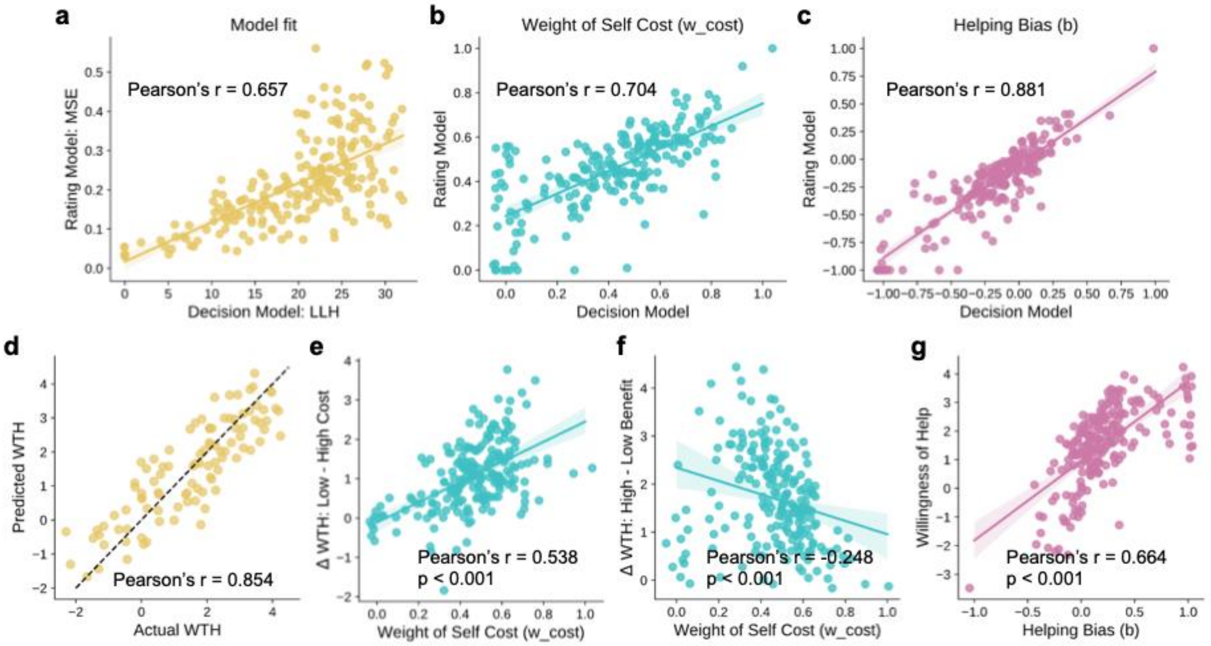

Supplementary Figure 5. Comparison between the binary decision model and the continuous rating model. A continuous version of the model 2.0 (rating model) that fit to the WTH ratings instead of binary choices (decision model) showed highly consistent (a) model fits (LLH: log-likelihood, MSE: mean squared error), (b) fitted parameter of w_{cost}, and (c) fitted parameter of *b* compared to the binary version. It also replicated multiple properties of the model, including (d) good out of sample prediction accuracy on the WTH ratings (replicating Figure 3f), (e) positive correlation between the weight of self cost (w_{cost}) and differences in WTH between low and high cost scenarios (replicating Figure 3c), (f) negative correlation between the weight of self cost (w_{cost}) and differences in WTH between high and low benefit scenarios (replicating Figure 3d), and (g) positive correlation between helping bias *b* (replicating Figure 3d) and WTH ratings (replicating Figure 3e). (a-c,e-g) display data from N = 215 participants, (d) displays data from N = 100 scenarios.

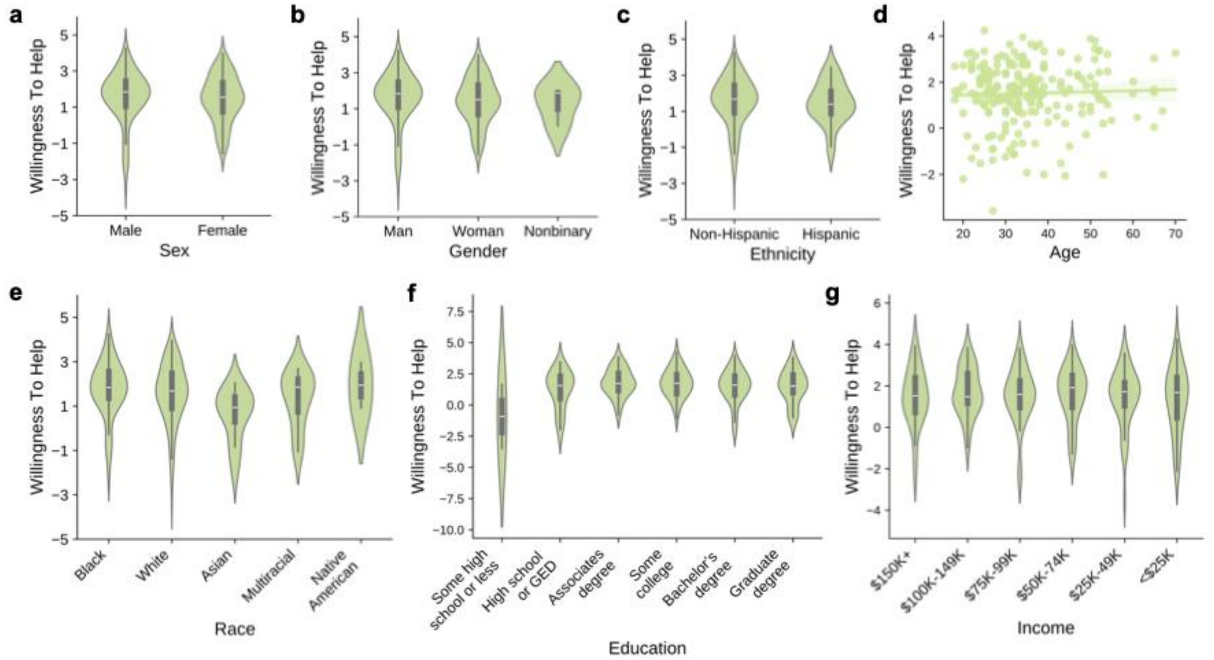

Supplementary Figure 6. Associations between willingness to help and demographic variables. (a) Biological sex. An independent sample Welch's t-test showed no statistically significant differences in WTH between male and females (T(207.7)=0.590, p=0.555, Cohen's d = 0.081, 95%CI = [-0.187, 0.348]). (b) Gender. A Welch's one-way ANOVA showed no statistically significant effect of gender on WTH (F(2,5.52) = 0.400, η^2= 0.004, p=0.688, 95%CI = [0,0.11]). (c) Ethnicity. An independent sample Welch's t-test showed no statistically significant effect of ethnicity in WTH (T(35.7)=0.204, Cohen's d = 0.037, 95% CI = [-0.373, 0.447], p=0.840). (d) Age. Pearson's correlation between age and WTH is not statistically significant (r = 0.038, p = 0.572, 95% CI = [-0.097, 0.167]). (e) Race. A Welch's one-way ANOVA showed no statistically significant effect of race on WTH (F(4,7.25)=2.60, p=0.12, η^2=0.05, 95%CI = [0,0.33]). (f) Education level. A Welch's one-way ANOVA showed no statistically significant effect of education level on WTH (F(5,10.6)=0.831, p =0.554, η^2=0.05, 95%CI = [0,0.28]). (g) Income level. A Welch's one-way ANOVA showed no statistically significant effect of income level on WTH (F(5,83.9)=0.354, p=0.878, η^2=0.009, 95%CI = [0,0.05]). The violin plots show kernel density estimate, median (white dot), 25th to 75th percentile (thick gray bar), and range of most extreme data points (think gray line). All the statistical analyses were performed on data of N=215 participants.

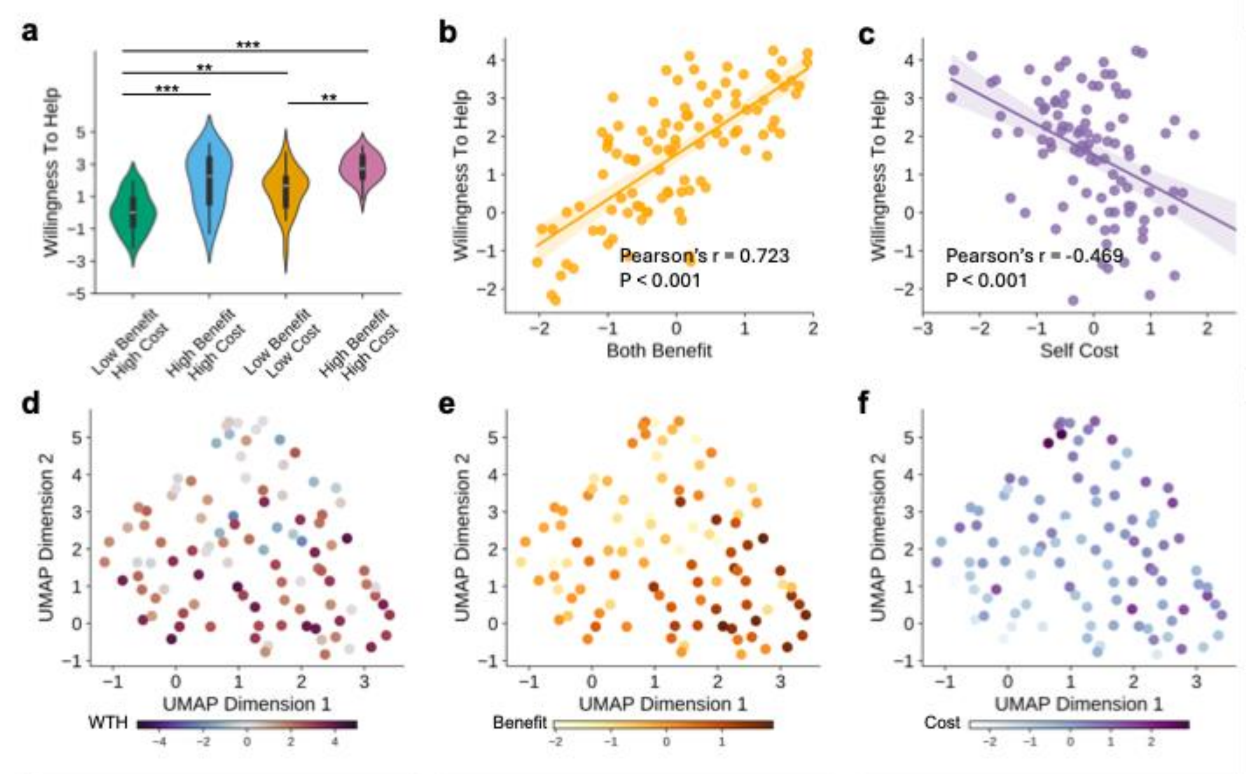

Supplementary Figure 7. Associations among three spaces. (a) Comparisons of the WTH among four motivation space quadrants. A one-way ANOVA suggested a significant main effect of motivation types on WTH ($F(3,34.7) = 34.6$, $p<0.001$). Post-hoc Games-Howell pairwise comparisons showed statistically significant differences between low benefit – high cost (n=27 scenarios) and high benefit – high cost quadrants (n=23 scenarios), low benefit – high cost and low benefit – low cost (n=23 scenarios) quadrants, low benefit – high cost and high benefit – high cost (n=27 scenarios) quadrants, low benefit – low cost and high benefit – high cost quadrants but not between high cost – high benefit and low cost – low benefit quadrants, and between high cost – high benefit and low cost – high benefit quadrants (see all stats in Supplementary Table 3). The violin plots show kernel density estimate, median (white dot), 25th to 75th percentile (thick gray bar), and range of most extreme data points (think gray line). (b) Correlation between WTH and Both Benefits. WTH significantly positively correlated with Both Benefits across scenarios (Pearson's r (98) = 0.723, $p<0.001$, 95% CI = [0.615,0.804]). (c) Correlation between WTH and Self Cost. WTH significantly negatively correlated with Both Benefits across scenarios (Pearson's r (98) = -0.469, $p<0.001$, 95% CI = [-0.597,-0.292]). (d) visualization of WTH in the semantic space. (e) visualization of Both Benefits in the semantic space. (f) visualization of Self Cost in the semantic space. Each dot represents one scenario (N=100 scenarios in total) in (b)-(f).

Rater	Scenario x	Scenario y	Scenario z
A	1	3	1
B	2	2	2
C	3	1	3
**Average**	2	2	2

→ WTH RDM

Less similar

→ residual RDM

More similar

Supplementary Figure 8. Decomposition of the decision similarity space. The decision similarity space quantifies two levels of similarity: similarity in consensus ratings of WTH (WTH RDM), and within-individual consistencies of ratings among scenarios (residual RDM). In this illustration, even though scenario x, y, and z all have an average rating of 2, scenario x is more similar to z than y because x and z are rated in the same way across three raters.

No.	Feature	Question	Choice	ICC
1	Empathy	How much do you empathize with the people in need?	Not at all - A great deal	0.931
2	Urgency	How urgent is this situation?	Not at all - A great deal	0.966
3	Descriptive norm	To what extent do you think your peers would help?	Definitely not - Definitely yes	0.921
4	Injunctive norm	To what extent would your peers think you should help?	Definitely not - Definitely yes	0.925
5	Self cost of help	Overall, how much it costs you to help?	Almost nothing - A great deal	0.890
6	Other's benefit of help	How much will the people in need benefit from being helped?	Not at all - A great deal	0.927
7	Indirect reciprocity	To what extent do you want to receive such help if you encounter a similar situation?	Not at all - Absolutely	0.923
8	Mood management	How much does this helping behavior improve your mood?	Not at all - A great deal	0.840
9	Reputation	How positively would this helping behavior improve your reputation?	Not at all - A great deal	0.890
10	Positive impact on self-concept	How much does this helping behavior improve your positive self-concept?	No change - A lot better	0.849
11	Self benefit of help	Overall, how is helping others in this situation good for yourself?	Not at all - A great deal	0.822
12	Other's cost of not help	How much bad consequence will the people in need experience?	Almost nothing - A great deal	0.963
13	Guilt	How much guilt do you feel?	Not at all - A great deal	0.947
14	Negative impact on self-concept	How much does not helping negatively influence your self-concept?	Not at all - A great deal	0.932
15	Self cost of not help	Overall, how is not helping in this situation bad for you?	Not at all - A great deal	0.925

Supplementary Table 1. Rating task questions. Participants rated on 15 questions during Survey 2. Each question ('Question') corresponds to a feature ('Feature') related to helping behaviors and was rated on 5-point Likert scales (see the two extremes in the 'Choice' column). All the questions achieved good to excellent inter-rater reliability ('ICC').

Model	k	R^2	AIC	BIC	Test Acc
1.0: Fixed bias	2	0.264	54.8	58.7	0.743
2.0: Single weight	3	0.420	**46.1**	**51.8**	**0.787**
3.0: Double weights	4	0.427	47.6	55.2	0.785
2.1: Nonlinear cost	4	0.422	48.0	55.6	0.763
2.2: Nonlinear benefit	4	0.411	48.7	56.3	0.690
2.3: Interactive	4	0.418	48.2	55.9	0.784
2.4: Urgency bonus	4	**0.441**	46.7	54.3	**0.787**

Supplementary Table 2. Model comparisons. Each of the seven candidate was fit to participants' data. The number of parameters (k), the group average pseudo R^2, Akaike Information Criteria (AIC), Bayesian Information Criteria (BIC), and model prediction accuracy on a held-out testing set (Test Acc) were compared among model candidates. The best performance model under each criterion was bolded.

Var 1	Var 2	M_{Var1}	M_{Var2}	diff	s.e.	T	df	*p*	Hedge's g
Low Benefit - High Cost	High Benefit - High Cost	0.03	1.96	-1.94	0.41	-4.68	37.96	<.001	-1.35
Low Benefit - High Cost	Low Benefit - Low Cost	0.03	1.37	-1.35	0.36	-3.80	43.87	<.001	-1.07
Low Benefit - High Cost	High Benefit - Low Cost	0.03	2.77	-2.74	0.27	-10.19	46.25	<.001	-2.73
High Benefit - High Cost	Low Benefit - Low Cost	1.96	1.37	0.59	0.45	1.32	41.81	.55	0.38
High Benefit - High Cost	High Benefit - Low Cost	1.96	2.77	-0.80	0.38	-2.10	30.28	.18	-0.62
Low Benefit - Low Cost	High Benefit - Low Cost	1.37	2.77	-1.40	0.32	-4.40	34.71	.00	-1.28

Supplementary Table 3. Post-hoc pairwise comparisons of WTH across four quadrants. Results from the Games-Howell post-hoc comparisons. The mean, mean difference, standard error of the difference of each pair of motivation types, as well as the T statistic, degrees of freedom (df), p value, and effect size (Hedges g) are displayed.

Motivation	Definition	Count	Example (Scenario #)
Contextual considerations	Situational factors would influence whether someone chooses to offer help, beyond personal traits or internal motivations	32	*Depends where they're stealing from (small business or corporate). (S29)*
Help could lead to bad consequences	Perceived outcome of help the person in need may not be positive and as expected, especially to the helpee	24	*Will more be expected of me than to just provide them a cleaning tool? Will they be alarmed at being approached? (S47)*
Prefer other options	A person prioritizes alternative actions over the current helping option	22	*I probably wouldn't help, they could just ask for directions. I don't want a stranger on my devices unless it's an emergency. (S4)*
Capacity	Perceived ability to help (is not enough)	21	*How will my shyness impact my ability to think up things to talk about? (S94)*
Not deserve help	The person in need is responsible for their own situation or has violated social/moral expectations	19	*I don't think anyone should allow themselves to be in that situation in the first place, and if they are, I think that there is a serious issue that helping them this one time will not solve. (S2)*
No need to help	There's "no need to help", even in a situation that seems like it could warrant it	14	*No one wants a stranger to come up and watch their young children for them anymore. You can't trust anyone. (S50)*
Similarity	Feels similar to the person in need, in the sense of connection, empathy, and group identity	3	*I suffer from panic attacks myself, and have physical limitations, but would definitely try to help someone in that situation regardless because I can relate to them. (S32)*
Responsibility	Believe it's someone else's job — especially someone more qualified, obligated, or officially designated to help	2	*A pet sitter needs to handle this. I would prefer not to get involved. (S48)*

Supplementary Table 4. Summary of self-reported additional motivations. Eight categories of motivations of helping (or not helping) identified from participant self-reports. Ranked in descending orders by the number of comments. Examples showed selected original open-ended response from the participants.
