# Supplementary material for: Prosocial decisions in naturalistic helping scenarios are predicted by cost-benefit tradeoffs and individual disposition
Source: Commun Psychol. 2025 Dec 20;4:2. doi: 10.1038/s44271-025-00371-x (PMC12770495; doi:10.1038/s44271-025-00371-x)
Supplement: Supplementary file 3 — Description of Additional Supplementary Files [file 44271_2025_371_MOESM3_ESM.docx]

**Description of Additional Supplementary Files**

File name- Supplemental Data 1

File description – The everyday helping scenarios stimulus set. Full descriptions of 100 everyday helping scenario narratives, prompt in Survey 1 and Survey 2, average willingness to help ratings, factor scores in the motivation space, and semantic space categories.
